# The relationship between urbanization and depression in China: the mediating role of neighborhood social capital

**DOI:** 10.1186/s12939-018-0825-x

**Published:** 2018-07-24

**Authors:** Ruoyu Wang, Desheng Xue, Ye Liu, Hongsheng Chen, Yingzhi Qiu

**Affiliations:** 1 School of Geography and Planning, Sun Yat-Sen University, Xingang Xi Road No.135, Guangzhou, 510275 China; 2 Guangdong Key Laboratory for Urbanization and Geo-simulation, Sun Yat-Sen University, Xingang Xi Road No.135, Guangzhou, 510275 China; 3 School of Architecture, Southeast University, Si-Pai-Lou Road No. 2, Nanjing, 210096 China

**Keywords:** Urbanization, Social capital, Depression, Neighborhood effect, China

## Abstract

**Background:**

Previous studies in developed countries have found that living in rapidly urbanizing areas is associated with higher risk of mental illness and that social capital had a protective effect on individual mental health. However, the literature is missing empirical studies of the relationship between urbanization, neighborhood social capital and mental health in rapidly urbanizing countries. To bridge this knowledge gap, this study investigated the effects of urbanization on depressive symptoms in China, with an emphasis on the mediating role of neighborhood social capital in the relationship between urbanization and individual-level depressive symptoms.

**Methods:**

Nationally representative survey data from the 2016 wave of China’s Labor-force Dynamics Survey were used. A sample of 20,861 individuals was obtained from 401 neighborhoods in 158 prefecture-level divisions of 29 provinces. Depressive symptoms were measured using CES-D scores. Neighborhood social capital was assessed by three individual-level variables aggregated to the neighborhood level: perceptions of neighborly trust, the extent of neighborly reciprocity, and membership to neighborhood social groups. Multilevel linear regression and mediation analyses were used to estimate the statistical relationships.

**Results:**

The multilevel linear regression analyses found negative relationships between urbanization rate and CES-D score. The mediation analysis found that neighborhood-level social capital was an inconsistent mediator in the relationship between urbanization rate and CES-D score. Interaction terms between urbanization rate and two measures of neighborhood-level social capital were statistically significant, indicating that the protective effects of neighborly reciprocity and membership to neighborhood social groups on CES-D scores (negative relationships) were stronger in the relatively more urbanized areas.

**Conclusion:**

Urbanization supports mental health in the Chinese context, although it might undermine residents’ mental health by reducing neighborhood social capital. The protective effect of neighborhood-level reciprocity and social group membership on mental health increased with urbanization.

## Background

China is experiencing unprecedented urbanization. In 1978, the proportion of the country’s urban population was almost 18%, which dramatically increased to more than 56% in 2015 [1]. It is estimated that more than one billion people will live in China’s cities by 2030 [2]. This rapid urbanization has evoked considerable scholarly interest in the effects of urbanization on individual mental health, particularly regarding symptoms of depression [3–6]. The prevalence of depression in China’s rapidly urbanizing areas poses a serious threat to the public health of cities. According to the 2015 Global Burden of Disease Study, depressive symptoms are the fifteenth leading cause of global disability-adjusted life years [7]. Moreover, depression is the main cause of disability, which increases mortality rates because depressed people are relatively more likely to commit suicide [8, 9].

A growing number of studies have suggested that urbanization might be detrimental to the mental health of urban residents [5, 6, 10, 11]. First, urban residents are more likely to be exposed to contextual stressors related to their physical environments such as traffic congestion, noise or air pollution, violence, and overcrowded living conditions, which might lead to discomfort and deteriorated psychological states [5, 6]. Second, urban residents are more likely to experience stressful personal events such as job loss, marital divorce or separation, mortgages, and residential relocation [12, 13]. Third, people living in rapidly urbanizing areas must adapt to new lifestyles—such as a faster pace of life and greater competition for jobs, goods, and services—which tend to increase stress [10, 11]. A few empirical studies have used cross-sectional survey data to validate this point by observing positive relationships between the extent of urbanization in China and residents’ depression [6, 14].

The role of social capital in mental health promotion has drawn increasing attention from public health scholars, policymakers, and practitioners. Numerous scholars have found that social capital has a protective effect on mental health [15–20]. Recent social epidemiological studies have investigated the effects of social capital operating at both micro (or individual) and macro (or ecological) levels [15]. At the micro level, people with relatively higher amounts of social capital are more able to tackle the negative effects of personal difficulties [21–23]. At the macro level, the collective social capital of a neighborhood is associated with the health of its residents, as social support from their neighbors helps them cope with the stress that arises from personal difficulties and experiences [19, 24–26].

Urbanization tends to bring strangers together, which might weaken kinship and friendship bonds and erode traditional social solidarity, and neighborhood social capital in urban areas might thereby be weakened [27]. Some studies on Western samples have found that people living in highly urbanized areas are relatively more likely to suffer from mental problems [15, 23, 28]. One reason for this phenomenon in the West is that urban residents tend to obtain relatively less social support from, and are more frequently involved in conflicts with, their neighbors [15, 23, 28]. Several researchers have argued that this association likely holds true in rapidly urbanizing countries because they are experiencing socio-demographic transitions—including decreasing proportions of extended families, increasing net migration rates, increasing proportions of single-parent households, and declining rates of fertility linked to increasing female participation in the labor force [10]. Thus, urbanization is surmised to negatively influence residents’ mental health by weakening neighborhood social capital, particularly in developing countries with rapid urbanization processes—such as China.

Although a growing body of literature has documented significant associations between urbanization and mental illness in China, few empirical studies have investigated the underlying mechanisms through which urbanization influences mental health, and there is scant research examining the protective effects of neighborhood social capital on mental health in China’s urbanizing areas. To bridge these gaps, this study investigated the relationship between urbanization and depressive symptoms in China using data from the 2016 wave of China’s Labor-force Dynamics Survey (CLDS 2016) [29]. The focus of this survey was on the mediating influence of neighborhood social capital in the relationship between urbanization and individual depressive symptoms. This study’s findings make two contributions to knowledge on the effects of urbanization on health in China. First, it specifies the association between the extent of urbanization and the perception of depression by exploring potential underlying mechanisms. Second, it enhances our understanding of mental health in China by considering the protective effects of neighborhood social capital on individual mental health.

### Data and methods

#### Data

This study used nationally representative survey data from the 2016 wave of the CLDS. Respondents were selected using a multistage, cluster, stratified Probability-Proportional-to-Size (PPS) sampling technique. The survey was carried out in the form of face-to-face interviews. In the first step, the survey team randomly chose 158 prefecture-level divisions from 29 provinces. Prefecture-level divisions are second-level administrative divisions that include prefectures, prefecture-level cities, and leagues. Then, 401 neighborhoods were randomly chosen from the prefecture-level divisions. Neighborhoods are China’s primary administrative divisions. The urbanization rates were derived from data in the China City Statistical Yearbook. After omitting cases missing essential data, the final dataset comprised 20,861 individuals in 401 neighborhoods in 158 prefecture-level divisions.

#### Variables

##### Dependent variable

The dependent variable was depressive symptoms, which were assessed using CES-D 20-item scores (Center for Epidemiology Studies of Depression 20-item). The CES-D 20-item scores include 20 questions measuring the mental state of residents over a week. These include experiences of feeling alone or disliked, and of people being unfriendly [30]. The Cronbach’s alpha of the 20 items was 0.95 in the overall sample.

##### Independent variables

Urbanization refers to the shifting of the population from rural to urban areas. Empirically, this study measured the urbanization rate as the ratio of usual urban residents to total population at the prefecture level in 2014 [5, 6, 31, 32]. The National Bureau of Statistics of China defines urban areas as the combination of city district areas (*cheng qu*) and township areas (*zhen qu*) [33]. City districts refer to both urban built-up areas where the seats of the municipal/district government are situated and their connective urban built-up areas. Townships refer to both urban built-up areas where the seats of the town government are situated and their connective urban built-up areas. Urban residents are defined as those who lived in urban areas for a consecutive period of six months [33]. This study calculated the urbanization rate of each prefecture of interest based on this official definition.

This study measured neighborhood-level social capital based on respondents’ answers to three items [15]: perception of neighborly trust, perception of neighborly reciprocity, and neighborhood social group memberships. Perception of neighborly trust was measured in the CLDS as the proportion of neighborhood residents that reported that they trusted those living in their neighborhoods. The CLDS measured neighborly reciprocity by the proportion of neighborhood residents who reported that they and their neighbors always or often helped one another. Social group membership was measured in the CLDS by the average number of voluntary group types within each neighborhood: *ju wei hui* (residents’ committee), social work organization, homeowners’ association, leisure/sports group, *tong xiang hui* (townsman association), clan organization, volunteer organization, and religious groups.

##### Control variables

The analyses controlled for the effects of personal characteristics previously found to co-vary with the key variables [8, 9, 34–36]. These variables are gender (dichotomy), age (continuous), marital status (categorical), educational attainment (categorical), employment status (dichotomy), *hukou* (residence status, dichotomy), urban/rural location (dichotomy), cigarette/cigar use (dichotomy), alcohol use (dichotomy), medical insurance status (dichotomy), physical health status (dichotomy), and weekly amount of physical exercise (continuous). The individual-level measures of social capital were (1) perception of whether neighbors are trustworthy (dichotomy), (2) perception of neighborly helpfulness (dichotomy), and (3) number of neighborhood social groups (dichotomy). Average annual household income per household member (continuous) and average annual neighborhood income per neighborhood resident (continuous) were also included. The mean VIF value was 1.44. Table 1 describes the variables used in this study’s analyses.

**Table 1:** Summary Statistics of Variables

Variable	Proportion/Mean (SD)
CES-D Score (0–60)	7.30 (9.24)
Urbanization rate (0–1)	0.52 (0.20)
Neighborhood-level social capital	
Neighborhood social trust (0–1)	0.78 (0.12)
Neighborhood social reciprocity (0–1)	0.48 (0.23)
Neighborhood social group membership (0–9)	0.08 (0.15)
Gender	
Male	48%
Female	52%
Age	44.83 (14.61)
Marital status	
Single, divorced, or widowed	19%
Married and living with spouse	73%
Married but not living with spouse	8%
Educational attainment	
Primary school or less	35%
High school	52%
College or more	13%
Employment	
Employed	95%
Unemployed	5%
*Hukou* status	
Local *hukou*	91%
Non-local *hukou*	9%
Urban/rural areas	
Urban areas	35%
Rural areas	65%
Smoking status	
Smoker	27%
Non-smoker	73%
Alcohol use	
Yes	19%
No	81%
Medical insurance	
Yes	90%
No	10%
Physical health status	
Disease	11%
No disease	89%
Physical exercise time (minutes per week)	97.51 (267.95)
Individual-level social capital	
Trust in neighbors	
Neighbors are extremely/very trustworthy	78%
Neighbors are somewhat/slightly/not at all trustworthy	22%
Neighbors are helpful	
Neighbors always/often help one another	48%
Neighbors sometimes/seldom/never help one another	52%
Number of voluntary group types	0.08 (0.37)
Average annual household income per household member (CNY)	17,991.68 (202,477.08)
Average annual neighborhood income per neighborhood resident (CNY)	17,814.06 (3.22)

## Methods

Multilevel models were superior to single-level models in this case because the CLDS data have a strong hierarchical structure. CES-D scores ranged from 0 to 60, and the statistical models tested the following equation.

$$ CES-{D}_{ihj}={\beta}_0+{\beta}_1 Social\kern0.17em capital\;{indicators}_{hj}+{\beta}_2 Urbanization\;{rate}_j+{\beta}_3 Co\mathit{\operatorname{var}}{iates}_{hj}+{\beta}_4 Co\mathit{\operatorname{var}}{iates}_{ihj}+{\varepsilon}_{ihj}+{\mu}_{hj}+{\upsilon}_j $$

In this equation, *i* indicates the individual, *h* represents the neighborhood, and *j* represents the prefecture-level division. *β*_*0*_ was the intercept. *Social capital indicators*_*hj*_ represented a vector of neighborhood-level measures of social capital. *Urbanization rate*_*j*_ represented a vector of prefecture-level urbanization rates. *Covariates*_*hj*_ and *Covariates*_*ihj*_ represented vectors of individual-level and neighborhood-level variables, respectively. In this equation, *ε*_*ihj*_*,* μ_hj_*, ν*_*j*_ represented the random error terms at the individual, neighborhood, and prefecture levels, respectively. The relationships of interest were assessed using the coefficients *β*_*1*_ and *β*_*2*_.

In addition, a mediation analysis examined the (inconsistent) mediation effects [37–40] of neighborhood-level social capital on the relationship between the urbanization rate at the prefecture level and individual-level CES-D scores.

## Results

Table 2 shows the results of Model 1, which included all the variables except for the neighborhood-level social capital variables. Urbanization rate negatively influenced CES-D score (coefficient = − 0.736, SE = 0.350). This indicates a significant negative relationship between the urbanization rate and respondents’ depressive symptoms. Males had lower CES-D scores than females (coefficient = − 1.245, SE = 0.153). CES-D scores increased with age (coefficient = 0.040, SE = 0.005). Married respondents had lower CES-D scores than those of other marital statuses (married and living with a spouse coefficient = − 1.056, SE = 0.179; married and not living with a spouse coefficient = − 0.719, SE = 0.264). Compared to respondents with primary education or less, respondents with a high school or college education or more had lower CES-D scores (high school coefficient = − 1.056, SE = 0.152; college or more coefficient = − 1.028, SE = 0.252). Employed respondents had lower CES-D scores than unemployed respondents (coefficient = − 0.557, SE = 0.265). Respondents with local *hukou* had lower CES-D scores (coefficient = − 0.555, SE = 0.265). Respondents with medical insurance had lower CES-D scores than those without medical insurance (coefficient = − 0.854, SE = 0.203), and those who reported disease had higher CES-D scores than those who reported good health (coefficient = 5.898, SE = 0.196). CES-D scores decreased with increased physical exercise time (coefficient = − 0.118, SE = 0.026).

**Table 2:** The relationships of urbanization rate and individual characteristics to CES-D scores; coefficient (standard error)

Variable	Model 1
Fixed effects	Coeff. (SE)
Urbanization rate	−0.736** (0.350)
Male (ref: female)	−1.245*** (0.153)
Age	0.040*** (0.005)
Marital status (ref. = single, divorced, or widowed)	
Married and living with a spouse	−1.056*** (0.179)
Married and not living with a spouse	−0.719*** (0.264)
Education (ref: primary school or less)	
High school	−1.056*** (0.152)
College or more	−1.028*** (0.252)
Employed (ref: unemployed)	−0.557** (0.265)
Local *hukou* (ref: non-local *hukou*)	−0.555** (0.265)
Urban areas (ref: rural areas)	−0.251(0.360)
Smoking status (ref: non-smoker)	0.100 (0.173)
Alcohol use (ref: no)	−0.059 (0.171)
Medical insurance (ref: none)	−0.854*** (0.203)
Physical health status (ref: no disease)	5.898*** (0.196)
Logarithm of physical exercise time	−0.118*** (0.026)
Logarithm of average annual household income per household member	−0.568*** (0.065)
Individual-level social capital	
Neighbors are extremely/very trustworthy (ref: neighbors are somewhat/slightly/not at all trustworthy)	−1.739*** (0.148)
Neighbors always/often help one another (ref: neighbors sometimes/seldom/never help one another)	−0.269** (0.112)
Number of voluntary group types	−1.139*** (0.131)
Logarithm of average annual neighborhood income per neighborhood resident	−0.961*** (0.329)
Constant	17.147*** (0.866)
Random effects	Coeff.
Var (Prefecture-level constant)	2.100***
Var (Neighborhood-level constant)	5.134***
Var (Residual)	70.300***
Number of prefectures	158
Number of neighborhoods	401
Number of respondents	20,861
Log-likelihood	−74,310
AIC	148,668

* = *p* < 0.10, ** = *p* < 0.05, *** = *p* < 0.01. All continuous independent variables and covariates were grand-mean centered

The CES-D score was negatively influenced by individual-level social capital: neighbors are extremely/very trustworthy (coefficient = − 1.739, SE = 0.148), neighbors always/often help one other (coefficient = − 0.269, SE = 0.112), and number of social groups (coefficient = − 1.139, SE = 0.131). Finally, CES-D scores were negatively influenced by the logarithm of average annual household income per household member and the average annual neighborhood income per neighborhood resident; these were coefficient = − 0.568 (SE = 0.065) and coefficient = − 0.961 (SE = 0.329), respectively.

Table 3 illustrates the effects of the urbanization rate on each of the three measures of neighborhood social capital (neighborly trust, neighborly reciprocity, and membership to neighborhood social groups). Models 2–4 show that the urbanization rate negatively influenced all three measures of neighborhood social capital (neighborly trust coefficient = − 0.172, SE = 0.039; neighborly reciprocity coefficient = − 0.244, SE = 0.072; and social group membership coefficient = − 0.113, SE = 0.031). This confirmed this study’s expectation that neighborhood social capital decreases with urbanization in China.

**Table 3:** The relationship of urbanization and individual characteristics to neighborhood social capital; coefficient (standard error)

Independent variable	Model 2: Neighborhood-level social trust	Model 3: Neighborhood-level social reciprocity	Model 4: Neighborhood-level social group membership
Fixed effects	Coeff. (SE)	Coeff. (SE)	Coeff. (SE)
Urbanization rate	−0.172*** (0.039)	− 0.244*** (0.072)	− 0.113*** (0.031)
Male (ref: female)	0.001 (0.001)	0.005** (0.002)	−0.007*** (0.002)
Age	0.000*** (0.000)	−0.001*** (0.000)	0.001*** (0.000)
Marital status (ref. = single, divorced, or widowed)			
Married and living with a spouse	−0.001 (0.001)	0.003 (0.002)	−0.004* (0.002)
Married and not living with a spouse	−0.002 (0.002)	0.002 (0.004)	−0.002 (0.003)
Education (ref: primary school or less)			
High school	−0.001 (0.001)	−0.020*** (0.002)	0.023*** (0.002)
College or more	0.001 (0.002)	−0.052*** (0.003)	0.054*** (0.003)
Employed (ref: unemployed)	−0.003 (0.002)	−0.007* (0.004)	0.002 (0.003)
Local *hukou* (ref: non-local *hukou*)	0.013*** (0.002)	0.030*** (0.003)	−0.002*** (0.001)
Urban areas (ref: rural areas)	−0.007 (0.006)	−0.144 (0.100)	− 0.214 (0.202)
Smoking status (ref: non-smoker)	−0.002 (0.001)	− 0.002 (0.002)	− 0.001 (0.002)
Alcohol use (ref: no)	0.001 (0.001)	−0.002 (0.002)	− 0.002 (0.002)
Medical insurance (ref: none)	−0.000 (0.002)	0.007** (0.003)	−0.006** (0.002)
Physical health status (ref: no disease)	−0.002 (0.002)	−0.010*** (0.003)	0.004* (0.002)
Logarithm of physical exercise time	−0.001*** (0.000)	− 0.004*** (0.000)	0.002*** (0.000)
Logarithm of average annual household income per household member	0.002*** (0.000)	−0.008*** (0.001)	0.011*** (0.001)
Individual-level social capital			
Neighbors are extremely/very trustworthy (ref: neighbors are somewhat/slightly/not at all trustworthy)	0.028*** (0.001)	0.004** (0.002)	0.002** (0.001)
Neighbors always/often help one another (ref: neighbors sometimes/seldom/never help one another)	0.004*** (0.001)	0.018*** (0.002)	0.089*** (0.002)
Number of types of voluntary groups	0.005*** (0.001)	0.072*** (0.002)	0.015*** (0.002)
Logarithm of average annual neighborhood income per neighborhood resident	0.003*** (0.001)	−0.045*** (0.002)	0.001*** (0.000)
Constant	0.860*** (0.021)	0.655*** (0.037)	−0.067*** (0.017)
Random effects	Coeff.	Coeff.	Coeff.
Var (prefecture-level constant)	0.008***	0.023***	0.005***
Var (residual)	0.004***	0.012***	0.007***
Number of prefectures	158	158	158
Number of respondents	20,861	20,861	20,861
Log-likelihood	26,536	15,849	22,164
AIC	−53,026	−31,652	−44,282

* = *p* < 0.10, ** = *p* < 0.05, *** = *p* < 0.01. All continuous independent variables and covariates were grand-mean centered

Table 4 shows the results of the multilevel linear regression analysis of the relationship of the urbanization rate and neighborhood-level social capital with the CES-D score. Model 5(a) included urbanization rate, all the individual-level and neighborhood-level variables, as well as the neighborhood-level measures of social capital as independent variables. Urbanization rate negatively influenced CES-D scores (coefficient = − 1.690, SE = 0.810), as did the three neighborhood-level social capital variables (social trust coefficient = − 3.503, SE = 1.473; social reciprocity coefficient = − 0.861, SE = 0.357; and social group membership coefficient = − 0.504, SE = 0.244). Compared to the corresponding coefficient in Model 1, the effect of the urbanization rate on CES-D scores was significantly stronger (in absolute terms) when the three neighborhood-level social capital variables were included in Model 5(a) (− 0.736 versus − 1.690, respectively). Neighborhood-level social capital was significantly and negatively related to CES-D scores, all of which passed the Sobel test. This finding suggests that the mediating effect of neighborhood-level social capital is inconsistent with respect to its influence on the relationship between urbanization rate and depressive symptoms [32].

**Table 4:** The effects of urbanization rate and individual characteristics on CES-D score mediated by neighborhood social capital; coefficient (standard error)

Variable	Model 5(a)	Model 5(b)
Fixed effects	Coeff. (SE)	Coeff. (SE)
Urbanization rate	−1.690** (0.810)	−1.547** (0.751)
Male (ref: female)	− 1.240*** (0.153)	− 1.239*** (0.153)
Age	0.040*** (0.005)	0.040*** (0.005)
Marital status (ref. = single, divorced, or widowed)		
Married and living with a spouse	−1.0539*** (0.179)	−1.053*** (0.179)
Married and not living with a spouse	− 0.724*** (0.264)	−0.725*** (0.264)
Education (ref: primary school or less)		
High school	−1.074*** (0.153)	−1.077*** (0.152)
College or more	−1.045*** (0.251)	−1.046*** (0.252)
Employed (ref: unemployed)	−0.553** (0.265)	−0.551** (0.265)
Local *hukou* (ref: non-local *hukou*)	−0.309 (0.248)	−0.319(0.248)
Urban areas (ref: rural areas)	−0.950(0.758)	−1.074(0.762)
Smoking status (ref: non-smoker)	0.093 (0.173)	0.093 (0.173)
Alcohol use (ref: none)	−0.057 (0.171)	−0.059 (0.17)
Medical insurance (ref: no)	−0.850*** (0.203)	−0.848*** (0.203)
Physical health status (ref: no disease)	5.886*** (0.196)	5.885*** (0.196)
Logarithm of physical exercise time	−0.120*** (0.026)	−0.120*** (0.026)
Logarithm of average annual household income per household member	−0.575*** (0.065)	−0.576*** (0.065)
Individual-level social capital		
Neighbors are extremely/very trustworthy (ref: neighbors are somewhat/slightly/not at all trustworthy)	−1.699*** (0.149)	−1.699*** (0.149)
Neighbors always/often help one another (ref: neighbors sometimes/seldom/never help one another)	−0.251*** (0.124)	−0.251*** (0.124)
Number of types of voluntary groups	−1.095*** (0.133)	−1.095*** (0.133)
Logarithm of average annual neighborhood income per neighborhood resident	−1.056** (0.3251)	−0.990** (0.326)
Neighborhood-level social capital		
Neighborhood social trust	−3.503** (1.473)	−3.952*** (1.535)
Neighborhood social reciprocity	−0.861** (0.357)	−0.719** (0.350)
Neighborhood social group membership	−0.504** (0.244)	−0.690** (0.299)
Cross-level interaction terms		
Neighborhood-level social trust × urbanization rate		−7.582 (7.582)
Neighborhood-level social reciprocity × urbanization rate		−1.415** (0.700)
Neighborhood-level social group membership × urbanization rate		−2.707** (1.350)
Constant	21.310*** (1.503)	21.637*** (1.541)
Random effects	Coeff.	Coeff.
Var (prefecture-level constant)	2.269***	2.434***
Var (neighborhood-level constant)	4.782***	4.651***
Var (residual)	70.298***	70.297***
Number of prefectures	158	158
Number of neighborhoods	401	401
Number of respondents	20,861	20,861
Log-likelihood	−74,303	−74,302
AIC	148,660	148,664

* = *p* < 0.10, ** = *p* < 0.05, *** = *p* < 0.01. All continuous independent variables and covariates were grand-mean centered

Model 5(b) added three cross-level interaction terms between the neighborhood-level social capital variables and urbanization rate to test whether the relationships between social capital and CES-D depended on the extent of urbanization. The results found that urbanization rate negatively moderated the relationships between social reciprocity and CES-D score, as well as between social group membership and CES-D score.

**Fig. 1:**
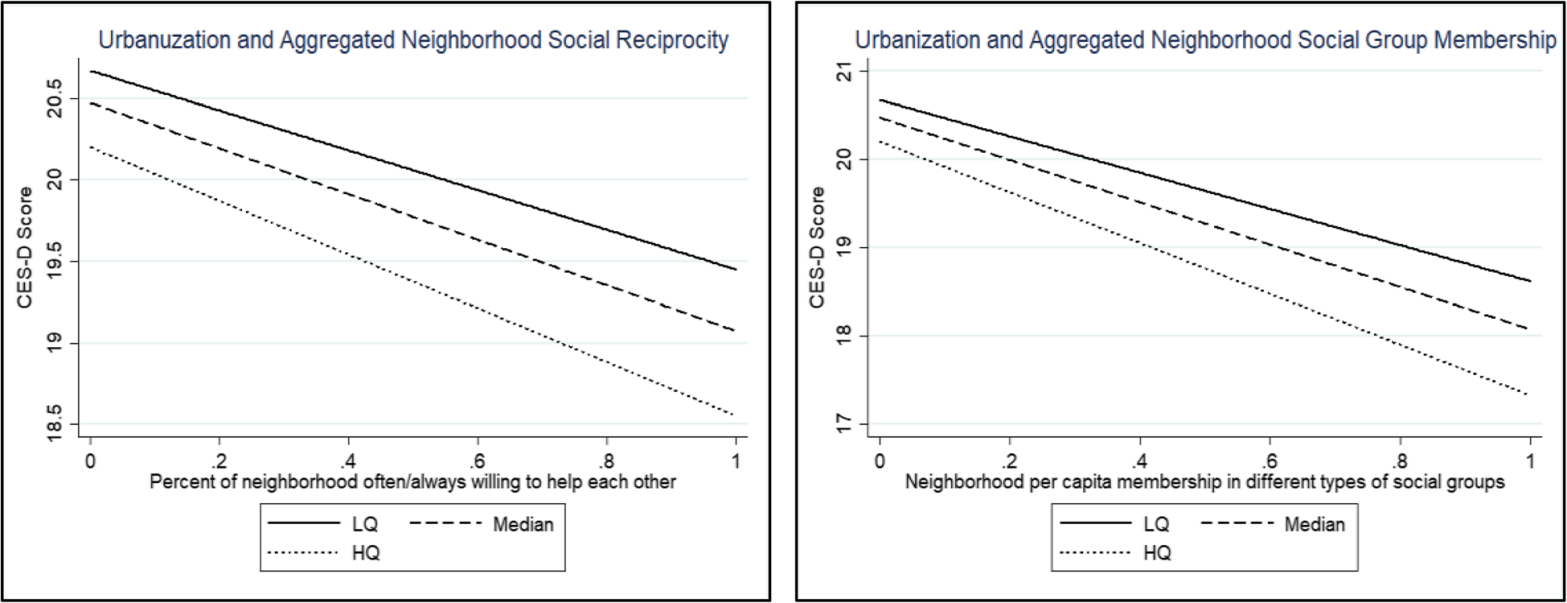
Predicted relationships between neighborhood social capital and CES-D scores by different level of urbanization: Low (25%), Median (50%), and High (75%)

Figure 1 illustrates the estimated effects of social reciprocity on CES-D scores, and the relationship between social group membership and CES-D scores for respondents living in urban areas. These areas are categorized into quartiles according to the extent of urbanization: Low (25%), Median (50%), and High (75%). Figure 1 suggests that the protective effects of neighborly reciprocity and neighborhood social group membership on the risk of depression (defined by the negative relationships of perceived reciprocity or social group membership to CES-D scores) were stronger in relatively more urbanized areas.

## Discussion

The novelty of this study is its consideration of neighborhood-level social capital in testing the effects of the urbanization rate on depressive symptoms in the Chinese context. The mediation analysis explored the possible underlying mechanisms by which urbanization influenced the respondents’ CES-D scores through three neighborhood-level social capital variables. The results found that urbanization negatively influenced depressive symptoms as expected, and that neighborhood-level social capital had an inconsistent mediation effect on the relationship between urbanization rate and depressive symptoms.

This study found that depression was less prevalent in relatively more urbanized areas, which is inconsistent with the results of previous studies on the mental health of people in China [5, 6]. This result might be explained by the healthy migration hypothesis; that is, the argument that relatively healthy and optimistic rural and small-town people are most likely to migrate to more urbanized areas, leaving behind those at higher risk of depression [41]. Another possible explanation is that residents living in less urbanized areas generally know less about depression and have fewer resources to cope with it than those in more urbanized areas, and are thus undertreated [42, 43]. Unlike residents living in more urbanized areas—who have better access to proper healthcare, including psychiatric treatment—residents living in less urbanized areas tend to have difficulty combating depression through formal means.

These findings further indicate that urbanization might erode neighborhood social capital, which may weaken the protective effect of social capital on the mental health of individuals. Rapid urbanization might weaken neighborhood social bonds for a variety of social and demographic reasons. First, as evidenced by Wellman’s research on communities in urbanizing areas, residents of relatively urban areas were more likely than non-urban residents to be involved in multiple social networks, although they rarely retained strong links to specific social networks [44]. Second, a high level of neighborhood turnover tends to undermine neighborhood social ties [45]. Furthermore, in addition to acting as a buffer between residents’ mental health and socioeconomic inequality [21], neighborhood social capital may speed up the diffusion of health-related information, promote residents’ access to health services, and regulate their unhealthy behaviors [15]. Thus, urbanization in China may contribute to the increase in the depressive symptoms of residents by eroding their neighborhood social capital.

This study found that urbanization enhanced the protective effect of neighborly reciprocity and social group membership on mental health. Several things might explain this result. First, residents living in more urbanized areas normally receive more instrumental and emotional support after they build close ties to their neighbors [5, 6, 31, 32]. Compared to residents living in less urbanized areas, those living in more urbanized areas generally have more resources and health knowledge to cope with stress and mental illness, enabling them to provide more effective support for their neighbors [5, 6, 31, 32].

Second, residents living in more urbanized areas are relatively more knowledgeable about depressive symptoms than their counterparts in less urbanized areas [42, 43]. As a result, they tend to be relatively more willing and able to access neighborhood social resources to counteract stressful life experiences and difficulties. However, this study found no evidence to suggest that urbanization significantly moderated the effects of trust among neighbors on mental health. The protective effect of trust among neighbors on mental health did not significantly vary according to urbanization rate. This is likely because trust is culturally embedded in China and its strength might not be easily influenced by changes in urban circumstances [45].

The relationship between the most significant control variables and depression is consistent with previous studies [8, 9, 34–36, 41–43, 46, 47]. Males had lower CES-D scores than females because the latter have more life pressures than males in China [41]. The CES-D scores increased with age, since elderly people are more vulnerable to life stresses than their younger counterparts [43, 46]. Married respondents had lower CES-D scores than those who are unmarried, divorced, or widowed, since married people normally suffer less from loneliness and the resultant depressive symptoms [43, 46]. Compared to respondents with primary education or less, respondents with a high school education or more had lower CES-D scores. This is because those with more education tend to have more health-related knowledge [42, 46]. Employed respondents had lower CES-D scores than unemployed respondents, since unemployment is a kind of life stressor [8, 9]. Local *hukou* holders had lower CES-D scores than their non-local *hukou* counterparts, as the former group can access more local medical services than the latter [42, 43, 46]. CES-D scores declined with increased physical exercise, since physical exercise is a good means of coping with stress [35, 36]. CES-D scores were negatively influenced by individual-level social capital because residents with more social capital are able to obtain support from others more easily [34]. Finally, CES-D scores were negatively influenced by the logarithm of average annual household income per household member and average annual neighborhood income per neighborhood. This is because residents with higher income and living in wealthier neighborhoods typically have access to more health knowledge and services than their poorer counterparts [42, 43].

From a policy perspective, this study suggests that greater efforts should be made to increase social capital in neighborhoods in the inevitable process of Chinese urbanization. First, policymakers should bring conflicting groups together and deliver on policy promises in order to encourage residents to trust in their relationships with one other [48]. Second, to encourage reciprocity among neighbors, policymakers should create and develop an ethos of community support by praising and rewarding those who help their neighbors [48]. Third, more public resources should be devoted to supporting local activities and local organization in order to encourage more residents to join neighborhood social groups [48]. Fourth, efforts should be made to promote a sense of belonging among neighborhood residents.

However, this study has four limitations. First, the results cannot support causal inferences between the extent of urbanization and depressive symptoms because the data were cross-sectional. Second, the results might be influenced by selection bias related to the effects of unobserved individual characteristics. For example, mentally healthier people might work and live in larger cities, while immigrants from the countryside and small towns may reverse-migrate when they begin to suffer from depressive symptoms. This could create an upward bias in estimates of the strength of the relationship between urbanization and mental health. Third, the neighborhoods analyzed in this study were defined as administrative units (community committees or village committees), and this may have created a modifiable areal unit problem in the estimation of the effects of neighborhood social capital on mental health [49, 50]. Lastly, the measurements of depressive symptoms and social capital were from respondents’ self-reports, and there could have been a gap between the respondents’ perceptions and objective reality.

## Conclusion

This study investigated the relationship between urbanization and depressive symptoms in China, with a particular focus on the mediating role of neighborhood-level social capital. Results of the multilevel linear regression analyses show that as urbanization increased, the risk of depression decreased. Results from mediation analysis indicate that neighborhood-level social capital has an inconsistent mediating effect on the relationship between urbanization and depressive symptoms. Finally, results regarding interaction effects found that the protective effects of neighborly reciprocity and social group membership on the risk of depression were stronger (had larger coefficients) in more urbanized areas than in less urbanized areas. These findings suggest that urbanization supports mental health in China, but that the effect is diminished when neighborhood social capital is weak in a context of rapid urbanization.

## Abbreviations

CES-D: Center of Epidemiological Survey-Depression Scale
CLDS: China Labor-force Dynamics Survey
